# Partner Ethnicity and Assisted Reproductive Technology Outcomes: A Retrospective Cohort Study

**DOI:** 10.3390/jcm14248962

**Published:** 2025-12-18

**Authors:** Shu Qin Wei, Michael H. Dahan, Yu Lu, Mingju Cao, Justin Tan, Seang Lin Tan

**Affiliations:** 1 Origin Elle Fertility Clinic & Women’s Health Centre, Montreal, QC H4A 3J3, Canada; shuqinwei@gmail.com (S.Q.W.); michael.dahan@mcgill.ca (M.H.D.); yu.lu7@mail.mcgill.ca (Y.L.); mcly3@hotmail.com (M.C.); 2 Department of Obstetrics and Gynecology, McGill University, Montreal, QC H3A 0G4, Canada; 3 CReATe Fertility Centre, Toronto, ON M5G 1N8, Canada; tan.justin@gmail.com

**Keywords:** assisted reproductive techniques, fertilization in vitro, ethnic groups, live birth, pregnancy, clinical, cohort studies

## Abstract

**Background:** Despite significant advances in assisted reproductive technology (ART), disparities in clinical outcomes persist. While patient-related factors are well-studied, the role of partner ethnicity remains understudied. We hypothesized that partner ethnicity affects ART outcomes. This study examined the association between partner ethnicity and ART outcomes. **Methods:** We conducted a retrospective cohort study among patients and their partners undergoing IVF treatment in the United Kingdom between 2017 and 2018. The exposure was partner ethnicity. Outcomes included biochemical pregnancy, clinical pregnancy, pregnancy loss, and live birth. We calculated risk ratios (RR) and 95% confidence intervals (CI) using multivariable regression models to estimate the association between partner ethnicity and IVF outcomes, adjusting for female patient age, partner age, patient ethnicity, gravidity, infertility diagnosis, treatment type, preimplantation genetic testing for aneuploidy, and number of prior in vitro fertilization (IVF) cycles. **Results:** Among 158,813 IVF cycles, live birth rates per cycle were 26.3% for couples with White partners and 23.1% for those with non-White partners. Non-White partners were associated with a 5% lower clinical pregnancy rate (RR 0.95, 95% CI 0.92–0.97) and a 6% lower live birth rate (RR 0.94, 95% CI 0.92–0.97). Specifically, Black (RR 0.82, 95% CI 0.77–0.87) and Asian (RR 0.67, 95% CI 0.59–0.76) partners had significantly reduced live birth rates, though these associations were attenuated after adjusting for patient ethnicity. Couples in which both the partner and patient were Black or Asian had 24–42% lower live birth rates compared with White couples (Black: RR 0.76, 95% CI 0.70–0.82; Asian: RR 0.58, 95% CI 0.49–0.68). **Conclusions:** Partner ethnicity is independently associated with IVF outcomes, with non-White partners showing lower rates of these outcomes. These findings suggest the clinical relevance of partner ethnicity in reproductive outcomes. Further research is warranted to elucidate the mechanisms underlying these associations.

## 1. Introduction

As global fertility rates continue to decline, the use of assisted reproductive technology (ART) has increased substantially, underscoring the need to understand the multifactorial determinants of ART treatment success [1,2]. Racial and ethnic disparities in ART outcomes among women are well established and consistently observed across different geographic regions [3,4]. In a U.S. cohort of 1646 frozen embryo transfer cycles, Black and Asian women had a 10–30% lower likelihood of live birth compared with White women [5]. Similarly, a U.K. study of 38,709 women reported that women of Black African and Bangladeshi descent had over 40% reduced odds of live birth following in vitro fertilization (IVF) or intracytoplasmic sperm injection (ICSI) compared with White women [6]. Despite these well-documented disparities, the potential influence of partner ethnicity on ART outcomes remains largely underexplored.

Emerging evidence suggests that semen parameters, such as sperm concentration, motility, and morphology, vary significantly across different ethnic groups [7,8,9]. In a U.K. cohort of 2996 infertile men, those of Sub-Saharan African and Asian descent had lower sperm motility than Caucasian men, and Asian men also had higher rates of sperm DNA fragmentation [7]. Another cohort of 3956 male patients from Canada found that African and Asian Canadians were more likely to be azoospermic and had lower semen volume compared with Caucasians [8]. Preliminary analyses of 1084 couples suggested that non-White men were more likely to have lower clinical pregnancy rate following IVF treatment [10]. Despite these observations, evidence regarding the influence of partner ethnicity on IVF success remains limited. We hypothesized that partner ethnicity may affect IVF outcomes. This study aimed to examine the association between partner ethnicity and ART outcomes in a large, population-based cohort.

## 2. Materials and Methods

### 2.1. Study Design and Population

**Figure 1:**
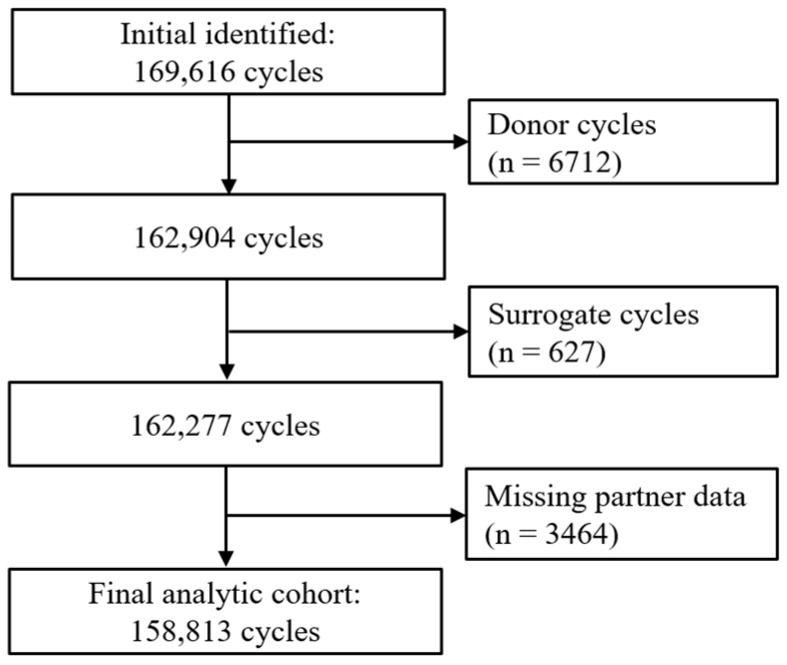
Flowchart of participant selection process.

We conducted a retrospective cohort study using anonymized data from the Human Fertilisation and Embryology Authority (HFEA) registry in the United Kingdom between January 2017 and December 2018 [11]. The HFEA maintains a comprehensive national registry of all licensed fertility treatment in the United Kingdom, with mandatory data submission by fertility clinics in accordance with UK legislation. The dataset comprised de-identified patient records. Eligible cases included patients and their partners who underwent ART during the study period. To ensure accurate assessment of partner ethnicity in relation to ART outcomes, we excluded 6712 cycles involving donors or single individuals without a recorded partner, 627 cycles with surrogates, and 3464 cycles with missing partner demographic information (Figure 1).

### 2.2. Exposure

The primary exposure was partner ethnicity, categorized as White, Black, Asian, or Other. White included White British, White Irish, and any other White background [11]. Black included Black African, Black Caribbean, and any other Black background [11]. Asian included Bangladeshi, Chinese, Indian, Pakistani, and any other Asian background [11]. Other encompassed Arab, Brazilian, Colombian, Iranian, Iraqi, Jewish, Vietnamese, individuals of mixed ethnic background, and all other ethnicities not classified above [11]. Ethnicity was generally self-reported and documented by clinical staff. The reference group was White partner ethnicity.

### 2.3. Outcomes

The main outcomes assessed in this study were biochemical pregnancy, clinical pregnancy, pregnancy loss, and live birth. A biochemical pregnancy was defined as a pregnancy diagnosed solely by the detection of beta human chorionic gonadotropin in blood or urine [12]. A clinical pregnancy was determined by the presence of one or more gestational sacs visualized via ultrasound or definitive clinical signs of pregnancy. Pregnancy loss encompassed any pregnancy that did not result in at least one live birth, irrespective of gestational duration or underlying cause [12]. A live birth was defined as the delivery of a fetus at or beyond 22 completed weeks of gestation which showed signs of life following separation from the mother [12].

### 2.4. Covariates

We considered maternal and partner factors that could confound the association be-tween partner ethnicity and ART outcomes, including female age at treatment (18–34, 35–39, ≥40 years), partner age at treatment (18–34, 35–39, ≥40 years), female patient ethnicity (White, Black, Asian, Other), gravidity (0, ≥1), infertility diagnosis (male factor, ovulatory disorder, tubal disease, endometriosis, unexplained), treatment type (ICSI, IVF), preimplantation genetic testing for aneuploidy (PGT-A, yes, no), and number of prior IVF cycles (0, 1, 2, ≥3). Key covariates, including infertility diagnosis, were documented by clinic staff based on clinical evaluation and standardized diagnostic criteria [11].

### 2.5. Statistical Analysis

We calculated descriptive characteristics of female patients and their partners, stratified by partner ethnicity. We also examined descriptive characteristics of cycles, including number of oocytes retrieved, embryos created and thawed, cycle type (fresh, frozen), stimulation used (yes, no), treatment type (ICSI, IVF), PGT-A (yes, no), number of previous cycles (0, 1, 2, ≥3), and use of elective single embryo transfer (eSET), all stratified by partner ethnicity.

We calculated outcome rates of biochemical pregnancy, clinical pregnancy, pregnancy loss, and live birth per 100 cycles across partner ethnic groups. In primary analysis, we used log-binomial regression models to estimate risk ratios (RRs) and 95% confidence intervals (CIs) for the association between partner ethnicity and IVF outcomes. We adjusted the models for female age, partner age, female patient ethnicity, gravidity, infertility diagnosis, and number of prior cycles. These covariates were selected a priori as key potential confounders based on their known influence on IVF success and the association with partner ethnicity. We used generalized estimating equations to account for intraclass correlation because patients could have more than one cycle.

Cycles with missing partner demographic information (2%) were excluded using complete-case analysis. We conducted sensitivity analyses by restricting to male partners only. We also examined ART outcomes among ethnically concordant couples (e.g., Black female and Black partner, Asian female and Asian partner) compared with White female–White partner couples. We performed data analyses using SAS software version 9.4 (SAS Institute Inc., Cary, NC, USA).

## 3. Results

### 3.1. Patient Characteristics

A total of 158,813 IVF cycles were included in the study, of which 3781 (2.4%) involved Black partners and 15,018 (9.5%) involved Asian partners (Table 1). Black partners were more likely than White partners to be ≥40 years at the time of treatment (50.3% vs. 34.5%). Most patients had partners of the same ethnicity: 90.9% of White patients had White partners, 86.2% of Asian patients had Asian partners, while 68.2% of Black patients had Black partners. Most partners were male, with proportions highest among Asian (99.3%) and Black (97.7%) groups compared to White partners (91.7%). Ovulatory disorders were more frequently reported as the cause of infertility among couples with Black (8.9%) and Asian partners (11.9%) than among those with White partners (7.7%). Tubal diseases were more frequently reported as the cause of infertility among couples with Black (13.3%) than those with White partners (7.2%).

### 3.2. Cycle Characteristics

The use of PGT-A was less common in couples with Black (0.7%) or Asian (1.1%) partners compared with those with White partners (1.2%) (Table 2). ICSI use was more frequent among couples with Black (40.1%) and Asian (39.7%) partners compared with those with White partners (37.4%). Fresh embryo transfer cycles were slightly less common in couples with Black (66.7%) and Asian (68.9%) partners compared with White partners (69.7%). eSET was also less common among Black (26.0%) and Asian (33.1%) partners compared with White partners (34.4%).

### 3.3. Association Between Partner Ethnicity and IVF Outcomes

Live birth rates per cycle were 26.3% among White partners and 23.1% among non-White partners (Table 3). After adjusting for female age, partner age, female ethnicity, gravidity, infertility diagnosis, and number of prior IVF cycles, non-White partners were associated with a 5% lower biochemical pregnancy rate (RR 0.95, 95% CI 0.93–0.97), a 5% lower clinical pregnancy rate (RR 0.95, 95% CI 0.92–0.97), and a 6% lower live birth rate (RR 0.94, 95% CI 0.92–0.97). However, non-White partners were not associated with pregnancy loss rate (RR 1.04, 95% CI 0.97–1.13).

When stratified by specific partner ethnicity, live birth rates per cycle were lower among couples with Black partners (20.4%) and Asian partners (23.2%) compared with White partners (26.3%) (Table 4). In models not adjusted for patient ethnicity, Black partners (RR 0.82, 95% CI 0.77–0.87) and Asian partners (RR 0.67, 95% CI 0.59–0.76) were associated with significantly lower live birth rates compared with White partners. These associations were attenuated after adjustment for female patient ethnicity (Black: RR 0.97, 95% CI 0.90–1.05; Asian: RR 0.94, 95% CI 0.80–1.11).

### 3.4. Sensitivity Analyses

Sensitivity analyses restricted to male partners yielded similar results (Supplemental Tables S1 and S2). Among couples in which both the patient and partner were Black, clinical pregnancy (RR 0.80, 95% CI 0.74–0.861) and live birth rates (RR 0.76, 95% CI 0.70–0.82) were 20–24% lower compared to White couples (Supplemental Table S3). Similarly, couples in which both partners were Asian had 36–42% lower clinical pregnancy (RR 0.64, 95% CI 0.55–0.73) and live birth rates (RR 0.58, 95% CI 0.49–0.68).

## 4. Discussion

In this large national cohort study of more than 158,000 IVF cycles, partner ethnicity was independently associated with ART outcomes. Black and Asian partners were more likely to be older at the time of treatment and to present with ovulatory disorders as a cause of infertility. Black and Asian partners underwent PGT-A less frequently than White partners and were also less likely to undergo eSET. Overall, non-White partners were associated with a 6% lower live birth rate. The most pronounced reductions were among racially concordant Black or Asian couples, who had 24–42% lower live birth rates compared with White couples.

Few studies have examined the influence of partner ethnicity on IVF outcomes. Prior research has primarily focused on semen analysis or general male characteristics [7,13]. A U.S. cohort of 615 men found that Black men had lower sperm concentration, total sperm count, percent motile sperm, and total motile sperm [13]. A cohort of 3956 male patients from Canada found that African Canadians and Asians had higher rates of azoospermia and lower volume compared with Caucasians [8]. A retrospective study involving 3484 participants found that men from Africa and Asian had poorer semen parameters compared with other regions [9]. In an unadjusted analysis, paternal race was associated with lower rates of clinical pregnancy among non-White men compared with White men [10]. In contrast, another analysis of 1878 IVF cycles reported no differences in early pregnancy outcomes between male racial groups. However, this study was limited by its single-center design and modest sample size [14].

Using data from a large, multicenter, population-based national cohort, our study extends the existing literature by showing that non-White partners were associated with lower clinical pregnancy and live birth rates in multivariable regression models, after adjusting for female ethnicity and other confounders. The greatest disparities were observed among concordant non-White couples. These findings suggest that partner ethnicity is a clinically relevant factor associated with disparities in reproductive outcomes. The exact mechanisms linking partner ethnicity to IVF outcomes remain incompletely understood. These disparities likely reflect a complex interplay of biological, clinical, environmental, and social determinants rather than intrinsic differences in fertility potential [3,4]. Differences in access to care, provider recommendations, and cultural preferences may partly explain the lower IVF success rates observed in non-White partners [3,4].

Our findings align with previous studies document ethnic disparities across the IVF pathway. Black and Asian patients have been reported to experience higher risks of cycle cancellation, failed fertilization, unintended freeze-all, and pregnancy loss compared with White patients [15]. A U.S. study reported that Black American women had higher odds of infertility due to anovulation or polycystic ovary syndrome compared with Black African women, as well as higher odds of tubal factor infertility [16]. These diagnoses are associated with reduced live birth rates and may partly explain disparities in ART outcomes [16]. In our study, Black and Asian partners were more likely to present with ovulatory disorders or tubal factor infertility, which may partly explain the reduced live birth rates.

Partner age likely contributes to these disparities. A cohort study of 6462 men undergoing fertility evaluation in Canada and the United States found that Black men were older at the time of assessment [17]. Similarly, in our study, Black and Asian partners were older and thus more likely to contribute to age-related reproductive risk. Advanced paternal age is consistently linked to decreased motility and increased sperm DNA fragmentation, affecting IVF outcomes [18,19]. A systematic review of 90 studies involving 94,000 male patients found that age-associated declines in semen motility, morphology, and unfragmented cells were statistically significant [20]. Another systematic review of 19 studies involving 40,668 men found that advancing paternal age was associated with an increased risk of DNA fragmentation. These findings suggest that age-related biological factors may contribute to the disparities we observed.

Differences in treatment characteristics may further explain outcome variability. We found lower use of PGT-A among Black and Asian partners compared with White partners. PGT-A is used to screen embryos for chromosomal aneuploidy to facilitate the selection of euploid embryos for transfer, but a Cochrane review found that there is insufficient high-quality evidence that it improves live birth rates after the first embryo transfer [21]. Black and Asian partners in our study were also less likely to undergo fresh embryo transfer, which has been associated with higher live birth rates than frozen transfer in certain populations [22]. Although these differences may act as potential confounders for lower live birth rates, we adjusted PGT-A and other covariates in our analyses, and the results remained consistent. Differences in PGT-A use may reflect inequities in access, provider recommendation patterns, or cultural preferences. Moreover, most ART protocols are developed in predominantly White populations, which may limit optimization for diverse ethnic groups. Historical data and current evidence do not support inherent fertility advantages for White couples, nor do they suggest that IVF is inherently more effective for White couples. Instead, protocol standardization based on predominantly White populations likely contributes to outcome differences. Future research in diverse populations, including the application of big data and artificial intelligence, may inform individualized protocols and improve equity.

Socioeconomic and structural factors likely contribute as well. Barriers such as limited insurance coverage, lower health literacy, and systemic biases in fertility care may influence treatment decisions, adherence, and outcomes [17]. Cultural factors and stress exposures may further compound reproductive disparities. The reduced live birth rates we observed among racially concordant Black and Asian couples highlight how shared sociodemographic and biological factors can interact to amplify risk. Our study found a reduction in rates of clinical pregnancy and live birth among racially concordant Black and Asian couples, suggesting potential compounded effects of shared sociodemographic or biological factors.

These findings have important clinical and research implications. Clinicians should consider partner ethnicity when counseling couples and tailoring treatment strategies. Careful attention to partner demographics, infertility diagnosis, and treatment history may help identify high-risk populations and optimize care pathways. Ensuring equitable access to technologies such as PGT-A and promoting eSET across diverse populations could help reduce disparities. Future research should systematically incorporate partner-level data and examine whether current IVF protocols, largely derived from White populations, adequately serve diverse ethnic groups by exploring the intersection of biological, environmental, and structural factors.

This study used a large, nationally representative registry, allowing for robust estimates of ART outcomes across diverse ethnic populations. Nevertheless, several limitations warrant consideration. Self-reported ethnicity may introduce non-differential misclassification, likely attenuating associations and underestimating the true effect sizes. Partner demographic data was missing for 2% of cycles, likely posing minimal selection bias, though slight under-representation cannot be excluded. As with most administrative datasets, coding errors and nondifferential misclassification may have occurred, likely attenuating associations. The dataset lacked information on potential confounders, including semen parameters, comorbidities, socioeconomic status, and lifestyle factors, which may influence the effect estimates. Socioeconomic disadvantages or other unmeasured confounders, which may be more prevalent in some ethnic groups, could influence treatment success and partly explain the observed associations. Data on clinics, geographic location, and environmental factors were unavailable, and clinic-level variability in protocols and reporting may have influenced exposure and outcome ascertainment. Finally, the retrospective, observational design is subject to residual confounding, precluding causal inference, and the findings may not generalize to settings without universal healthcare or with different ethnocultural distributions.

## 5. Conclusions

In this large national cohort study of over 158,000 IVF cycles, partner ethnicity was independently associated with key clinical outcomes. Couples with non-White partners, particularly those with Black or Asian partners, experienced significantly lower rates of clinical pregnancy and live birth. Black and Asian partners were older at the initiation of treatment and less likely to undergo PGT-A, factors that may contribute to reduced success rates. These findings suggest the clinical relevance of partner ethnicity in ART outcomes and support the value of considering both partners in future research. Further investigation is warranted to clarify the mechanisms underlying these differences and to guide strategies aimed at optimizing success rates across diverse patient populations.

## Figures and Tables

**Table 1:** Characteristics of female patients and their partners, stratified by partner ethnicity.

	No. Cycles (%)			
	White (n = 106,920)	Black (n = 3781)	Asian (n = 15,018)	Other (n = 33,094)
Partner age, years				
18–34	34,798 (32.6)	772 (20.4)	4965 (33.1)	7643 (23.1)
35–39	35,175 (32.9)	1106 (29.3)	5441 (36.2)	9032 (27.3)
≥40	36,947 (34.5)	1903 (50.3)	4612 (30.7)	16,419 (49.6)
Female age, years				
18–34	44,785 (41.9)	1410 (37.3)	7498 (49.9)	12,475 (37.7)
35–39	40,605 (38.0)	1352 (35.8)	5127 (34.2)	12,829 (38.8)
≥40	21,530 (20.1)	1019 (26.9)	2393 (15.9)	7790 (23.5)
Female ethnicity				
White	97,145 (90.9)	828 (21.9)	1299 (8.7)	10,011 (30.2)
Black	924 (0.8)	2577 (68.2)	30 (0.2)	455 (1.4)
Asian	2525 (2.3)	78 (2.0)	12,947 (86.2)	1193 (3.6)
Other	6326 (5.9)	298 (7.9)	742 (4.9)	21,435 (64.8)
Partner type				
Male	98,075 (91.7)	3695 (97.7)	14,907 (99.3)	32,309 (97.6)
Female	8845 (8.3)	86 (2.3)	111 (0.7)	785 (2.4)
Gravidity				
0	86,850 (81.2)	3141 (83.1)	12,463 (83.0)	27,010 (81.6)
≥1	20,070 (18.8)	640 (16.9)	2555 (17.0)	6084 (18.4)
Infertility diagnosis				
Male factor	36,767 (34.4)	1289 (34.1)	4930 (32.8)	8688 (26.2)
Ovulatory disorder	8197 (7.7)	337 (8.9)	1791 (11.9)	2164 (6.5)
Tubal disease	7661 (7.2)	501 (13.3)	928 (6.2)	1847 (5.6)
Endometriosis	3675 (3.4)	102 (2.7)	524 (3.5)	946 (2.9)
Unexplained	50,620 (47.3)	1552 (41.0)	6845 (45.6)	19,449 (58.8)

**Table 2:** Cycle characteristics stratified by partner ethnicity.

	No. Cycles (%)			
	White (n = 106,920)	Black (n = 3781)	Asian (n = 15,018)	Other (n = 33,094)
Oocytes retrieved				
0	45,528 (42.6)	1636 (43.3)	5709 (38.0)	14,515 (43.9)
1 to 5	16,565 (15.5)	668 (17.7)	2434 (16.2)	4919 (14.9)
6 to 10	20,375 (19.1)	595 (15.7)	3025 (20.1)	5633 (17.0)
11 to 15	13,506 (12.6)	451 (11.9)	2086 (13.9)	4175 (12.6)
≥16	10,946 (10.2)	431 (11.4)	1764 (11.8)	3852 (11.6)
Total embryo created				
0	46,072 (43.1)	1717 (45.4)	6197 (41.3)	16,216 (49.0)
1 to 5	32,502 (30.4)	1192 (31.5)	4750 (31.6)	8762 (26.5)
≥6	28,346 (26.5)	872 (23.1)	4071 (27.1)	8116 (24.5)
Total embryos thawed				
0	74,389 (69.6)	2520 (66.6)	10,318 (68.7)	22,843 (69.0)
1 to 5	31,858 (29.8)	1231 (32.6)	4556 (30.3)	10,102 (30.5)
≥6	673 (0.6)	30 (0.8)	144 (1.0)	149 (0.5)
Cycle types				
Fresh cycle	74,565 (69.7)	2523 (66.7)	10,346 (68.9)	22,896 (69.2)
Frozen cycle	32,355 (30.3)	1258 (33.3)	46,72 (31.1)	10,198 (30.8)
Stimulation used				
Yes	65,329 (61.1)	2196 (58.1)	9570 (63.7)	20,035 (60.5)
No	41,591 (38.9)	1585 (41.9)	5448 (36.3)	13,059 (39.5)
Treatment type				
ICSI	39,973 (37.4)	1590 (40.1)	5966 (39.7)	13,154 (39.8)
IVF	66,947 (62.6)	2191 (57.9)	9052 (60.3)	19,940 (60.2)
eSET				
Yes	36,823 (34.4)	982 (26.0)	4963 (33.1)	9234 (27.9)
No	70,097 (65.6)	2799 (74.0)	10,055 (66.9)	23,860 (72.1)
PGT-A treatment				
Yes	1322 (1.2)	28 (0.7)	161 (1.1)	407 (1.2)
No	105,598 (98.8)	3753 (99.3)	14,857 (99.9)	32,687 (98.8)
Number of prior cycles				
0	44,453 (41.6)	1558 (41.2)	6056 (40.3)	13,851 (41.9)
1	25,983 (24.3)	980 (25.9)	3905 (26.0)	7821 (23.6)
2	15,451 (14.5)	567 (15.0)	2260 (15.1)	4803 (14.5)
≥3	21,033 (19.6)	676 (17.9)	2797 (18.6)	6619 (20.0)

Note: IVF = in vitro fertilization; ICSI = intracytoplasmic sperm injection; eSET = elective single embryo transfer; PGT-A = preimplantation genetic testing for aneuploidy.

**Table 3:** Association between partner ethnicity and in vitro fertilization (IVF) outcomes (Non-White vs. White).

	Total No.	No. Events	Rate per 100 Cycles	Model 1 ^a^	Model 2 ^b^	Model 3 ^c^
Biochemical pregnancy						
White	106,920	37,728	35.3	1	1	1
Non-White	51,893	16,474	31.7	0.90 (0.89–0.91)	0.92 (0.91–0.94)	0.95 (0.93–0.97)
Clinical pregnancy						
White	106,920	31,721	29.7	1	1	1
Non-White	51,893	13,749	26.5	0.89 (0.88–0.91)	0.92 (0.90–0.93)	0.95 (0.92–0.97)
Pregnancy loss						
White	106,920	3589	3.4	1	1	
Non-White	51,893	1885	3.6	1.08 (1.02–1.14)	1.09 (1.03–1.15)	1.04 (0.97–1.13)
Live birth						
White	106,920	28,084	26.3	1	1	
Non-White	51,893	12,011	23.1	0.88 (0.86–0.90)	0.91 (0.89–0.92)	0.94 (0.92–0.97)

^a^ Model 1: Univariate regression assessing the association between partner ethnicity (Non-White vs. White) and IVF outcomes. ^b^ Model 2: Multivariable regression estimating the risk ratio for non-White vs. White partners, adjusted for female age, partner age, gravidity, infertility diagnosis, treatment type, preimplantation genetic testing for aneuploidy, and number of prior IVF cycles. ^c^ Model 3: Model 2 with additional adjustment for patient ethnicity.

**Table 4:** Association between partner ethnicity and in vitro fertilization (IVF) outcomes (Black, Asian, Other vs. White).

	Total No.	No. Events	Rate Per 100 Cycles	Model 1 ^a^	Model 2 ^b^	Model 3 ^c^
Biochemical pregnancy						
White	106,920	37,728	35.3	1	1	1
Black	3781	1070	28.3	0.80 (0.76–0.84)	0.84 (0.80–0.88)	0.96 (0.90–1.03)
Asian	15,018	4871	32.4	0.60 (0.53–0.68)	0.70 (0.64–0.78)	0.93 (0.82–1.05)
Other	33,094	10,533	31.8	0.47 (0.38–0.56)	0.59 (0.51–0.69)	0.89 (0.74–1.08)
Clinical pregnancy						
White	106,920	31,721	29.7	1	1	1
Black	3781	906	24.0	0.81 (0.76–0.86)	0.85 (0.80–0.90)	0.97 (0.90–1.04)
Asian	15,018	4006	26.7	0.60 (0.53–0.68)	0.72 (0.64–0.80)	0.94 (0.81–1.09)
Other	33,094	8837	26.7	0.47 (0.38–0.56)	0.61 (0.51–0.72)	0.91 (0.73–1.14)
Pregnancy loss						
White	106,920	3270	3.1	1	1	1
Black	3781	124	3.3	1.07 (0.90–1.28)	1.13 (0.96–1.34)	1.14 (0.92–1.42)
Asian	15,018	480	3.2	1.15 (0.81–1.64)	1.29 (0.93–1.79)	1.31 (0.84–2.03)
Other	33,094	1079	3.3	1.23 (0.73–2.09)	1.46 (0.89–2.39)	1.49 (0.77–2.89)
Live birth						
White	106,920	28,084	26.3	1	1	1
Black	3781	770	20.4	0.78 (0.73–0.83)	0.82 (0.77–0.87)	0.97 (0.90–1.05)
Asian	15,018	3489	23.2	0.60 (0.53–0.68)	0.67 (0.59–0.76)	0.94 (0.80–1.11)
Other	33,094	7752	23.4	0.47 (0.38–0.56)	0.55 (0.46–0.66)	0.92 (0.72–1.16)

^a^ Model 1: Univariate regression assessing the association between partner ethnicity (Black, Asian, Other vs. White) and IVF outcomes. ^b^ Model 2: Multivariable regression estimating the risk ratio for Black, Asian, Other vs. White partners, adjusted for female age, partner age, gravidity, infertility diagnosis, treatment type, preimplantation genetic testing for aneuploidy, and number of prior IVF cycles. ^c^ Model 3: Model 2 with additional adjustment for patient ethnicity.

## Data Availability

The original contributions presented in this study are included in the article/Supplementary Materials. Further inquiries can be directed to the corresponding author.

## Supplementary Materials

The following supporting information can be downloaded at: https://www.mdpi.com/article/10.3390/jcm14248962/s1, Table S1. Sensitivity analysis of male partner ethnicity (Non-White vs. White) and in vitro fertilization (IVF) outcomes; Table S2. Sensitivity analysis of male partner ethnicity and in vitro fertilization (IVF) outcomes; Table S3. Association of couple ethnicity with in vitro fertilization (IVF) outcomes.
