# Supplementary material for: Partner Ethnicity and Assisted Reproductive Technology Outcomes: A Retrospective Cohort Study
Source: J Clin Med. 2025 Dec 18;14(24):8962. doi: 10.3390/jcm14248962 (PMC12733499; doi:10.3390/jcm14248962)
Supplement: Supplementary file 1 [file jcm-14-08962-s001.zip › jcm-4015591-supplementary.pdf]

Journal of
Clinical Medicine

MDPI

Supplementary Table S1. Sensitivity analysis of male partner ethnicity (Non-White vs. White) and in vitro fertilization (IVF) outcomes.

	Total no.	No. Events	Rate per 100 Cycles	Model 1 [a]	Model 2 [b]	Model 3 [c]
Biochemical pregnancy						
White	98,075	35,066	35.8	1	1	1
Non-White	50,911	16,196	31.8	0.90 (0.89–0.91)	0.91 (0.90–0.93)	0.94 (0.92–0.96)
Clinical pregnancy						
White	98,075	29,432	30.0	1	1	1
Non-White	50,911	13,515	26.5	0.88 (0.87–0.90)	0.91 (0.89–0.92)	0.94 (0.92–0.96)
Pregnancy loss						
White	98,075	3328	3.4	1	1	1
Non-White	50,911	1858	3.6	1.08 (1.02–1.14)	1.09 (1.03–1.15)	1.04 (0.96–1.12)
Live birth						
White	98,075	26,002	26.5	1	1	1
Non-White	50,911	11,815	23.2	0.88 (0.86–0.89)	0.90 (0.88–0.92)	0.94 (0.91–0.96)

[a] Model 1: Univariate regression assessing the association between partner ethnicity (Non-White vs. White) and IVF outcomes.
[b] Model 2: Multivariable regression estimating the risk ratio for non-White vs. White partners, adjusted for female age, partner age, gravidity, infertility diagnosis, treatment type, preimplantation genetic testing for aneuploidy, and number of prior IVF cycles.

[c] Model 3: Model 2 with additional adjustment for patient ethnicity.

Supplementary Table S2. Sensitivity analysis of male partner ethnicity and in vitro fertilization (IVF) outcomes.

	Total No.	No. Events	Rate per 100 Cycles	Model 1 [a]	Model 2 [b]	Model 3 [c]
Biochemical pregnancy						
White	106,920	37,728	35.3	1	1	1
Black	3781	1070	28.3	0.80 (0.76–0.84)	0.83 (0.79–0.88)	0.96 (0.90–1.02)
Asian	15,018	4871	32.4	0.60 (0.53–0.68)	0.69 (0.62–0.77)	0.92 (0.81–1.05)
Other	33,094	10,533	31.8	0.47 (0.38–0.56)	0.58 (0.49–0.67)	0.88 (0.72–1.07)
Clinical pregnancy						
White	106,920	31,721	29.7	1	1	1
Black	3781	906	24.0	0.81 (0.76–0.86)	0.84 (0.79–0.89)	0.97 (0.90–1.04)
Asian	15,018	4006	26.7	0.60 (0.53–0.68)	0.71 (0.63–0.79)	0.94 (0.81–1.09)
Other	33,094	8837	26.7	0.47 (0.38–0.56)	0.59 (0.50–0.71)	0.91 (0.73–1.14)
Pregnancy loss						
White	106,920	3270	3.1	1	1	1
Black	3781	124	3.3	1.07 (0.90–1.28)	1.15 (0.97–1.36)	1.16 (0.93–1.46)
Asian	15,018	480	3.2	1.15 (0.81–1.64)	1.32 (0.95–1.84)	1.35 (0.87–2.12)
Other	33,094	1079	3.3	1.23 (0.73–2.09)	1.52 (0.92–2.49)	1.58 (0.81–3.08)

Live birth						
White	106,920	28,084	26.3	1	1	1
Black	3781	770	20.4	0.78 (0.73–0.83)	0.82 (0.77–0.87)	0.98 (0.90–1.06)
Asian	15,018	3489	23.2	0.60 (0.53–0.68)	0.67 (0.59–0.76)	0.96 (0.81–1.13)
Other	33,094	7752	23.4	0.47 (0.38–0.56)	0.55 (0.45–0.66)	0.94 (0.73–1.20)

[a] Model 1: Univariate regression assessing the association between partner ethnicity (Black, Asian, Other vs. White) and IVF outcomes.

[b] Model 2: Multivariable regression estimating the risk ratio for Black, Asian, Other vs. White partners, adjusted for female age, partner age, gravidity, infertility diagnosis, treatment type, preimplantation genetic testing for aneuploidy, and number of prior IVF cycles.

[c] Model 3: Model 2 with additional adjustment for patient ethnicity.

Supplementary Table S3. Association of couple ethnicity with in vitro fertilization (IVF) outcomes.

	**Total No.**	**No. Event**	**Rate per 100 Cycles**	**Unadjusted RR**	**Adjusted RR [a]**
Biochemical pregnancy					
White	97145	34751	35.8	1	1
Black	2577	694	26.9	0.75 (0.71–0.80)	0.80 (0.75–0.85)
Asian	12947	4211	32.5	0.57 (0.50–0.64)	0.64 (0.56–0.72)
Clinical pregnancy					
White	97145	29249	30.1	1	1
Black	2577	582	22.6	0.75 (0.70–0.81)	0.80 (0.74–0.86)
Asian	12947	3452	26.7	0.56 (0.49–0.65)	0.64 (0.55–0.73)
Pregnancy loss					
White	97145	3017	3.1	1	1
Black	2577	91	3.5	1.15 (0.95–1.40)	1.17 (0.96–1.42)
Asian	12947	433	3.3	1.33 (0.90–1.96)	1.37 (0.93–2.02)
Live birth					
White	97145	25944	26.7	1	1
Black	2577	487	18.9	0.71 (0.65–0.77)	0.76 (0.70–0.82)
Asian	12947	3004	23.2	0.50 (0.43–0.59)	0.58 (0.49–0.68)

[a] Multivariable regression estimating the risk ratio for Black, Asian vs. White partners, adjusted for female age, partner age, gravidity, infertility diagnosis, treatment type, preimplantation genetic testing for aneuploidy, and number of prior IVF cycles.
